# Screen time duration and timing: effects on obesity, physical activity, dry eyes, and learning ability in elementary school children

**DOI:** 10.1186/s12889-021-10484-7

**Published:** 2021-02-28

**Authors:** Yui Mineshita, Hyeon-Ki Kim, Hanako Chijiki, Takuya Nanba, Takae Shinto, Shota Furuhashi, Satoshi Oneda, Mai Kuwahara, Anzu Suwama, Shigenobu Shibata

**Affiliations:** 1 Laboratory of Physiology and Pharmacology, School of Advanced Science and Engineering, Waseda University, 2-2 Wakamatsu-cho Shinjuku, Tokyo, 162-0056 Japan; 2 Graduate School of Advanced Science and Engineering, Waseda University, 2-2 Wakamatsu-cho Shinjuku, Tokyo, 162-0056 Japan

**Keywords:** Screen time, Elementary school students, Obesity, Physical activity, Dry eyes, Learning ability

## Abstract

**Background:**

As internet use becomes more widespread, the screen time (ST) of elementary school students increases yearly. It is known that longer durations of ST can affect obesity, physical activity, dry eye disease, and learning ability. However, the effects of ST just before bedtime have not been clarified. Therefore, we examined ST duration and timing effects on elementary school children.

**Methods:**

We conducted a survey of 7419 elementary school students in Tokyo, Japan using a questionnaire on food education. ST duration and timing (just before bedtime) served as the explanatory variables, and the relationship between obesity, physical activity, dry eyes, and learning ability was analyzed using logistic regression analysis. Gender, school year, height, and weight were considered confounding factors. First, we examined whether ST duration and timing were related to each objective variable, using a univariate model to examine all variables. Thereafter, we performed multivariate logistic regression analyses for all variables showing a significant difference in the univariate models.

**Results:**

A significant association was observed between ST duration and obesity, physical activity, and academic performance, indicating that a longer ST duration may lead to obesity, decreased physical activity, and decreased academic performance. ST timing was associated with obesity, dry eyes, and academic performance, and ST immediately before bedtime contributed to obesity, dry eyes, and reduced academic performance. Furthermore, the results of investigating the combined effect of ST duration and timing (immediately before bedtime) on these factors revealed that ST timing has a greater effect on dry eyes, and ST duration has a greater effect on academic performance.

**Conclusion:**

Our findings indicate that ST in school children is related to obesity, physical activity, dry eyes, and learning ability, and they suggest that not only the duration but also the timing of ST is important.

**Supplementary Information:**

The online version contains supplementary material available at 10.1186/s12889-021-10484-7.

## Background

The increasing levels of internet access indicates steadily increasing usage time (screen time or ST) of digital devices such as TVs, smartphones, tablets, and gaming consoles increases year after year. According to data from a survey of elementary school students conducted by the Mobile Marketing Data Laboratory, 40.1% of students began using smartphones in 2019 [1]. In addition, data from the Cabinet Office of Japan indicate that in 2018, the internet usage rate exceeded 85% for elementary school students, and the average internet usage time increased by approximately 21 min compared to that in the previous year [2]. These data suggest that children are going online at younger ages and that elementary school students’ ST is increasing.

The various effects of longer ST duration on children’s health and life events are widely known. In the physical activity guidelines for children, ST is positively associated with sedentary behavior, therefore it is recommended to be limited to 2 h a day [3]. The World Health Organization (WHO) recommends that children and adolescents (5–17 years of age) perform at least 60 min of moderate to vigorous physical activity per day [4]. Nevertheless, in Japan, it was found that approximately 60% of children exceeded the two-hour-per-day maximum of sedentary behavior [5], and approximately half of the Japanese elementary school students did not achieve the recommended physical activity levels [6]. Lower levels of physical activity and longer sedentary periods from longer ST duration can increase the risk of obesity. Additionally, recent studies have demonstrated the potential effects of sedentary behavior and physical activity on academic performance [3, 7, 8], and that long periods of focused ST may lead to incomplete eye blinking and therefore, may result in dry eyes [9–11]. However, few studies have examined the relationship between ST and dry eye disease in children.

Along with the duration of the ST, the time of ST occurrence (e.g., just before bedtime) is also an important factor in ST-induced decreased physical activity, academic performance, and the increased frequency of dry eye disease. Visible light affects the central biological clock in the suprachiasmatic nucleus of the hypothalamus in the human brain, with morning light exposure advancing this biological clock and late-night light exposure (including light from LED devices) delaying it [12]. In addition, nighttime light exposure suppresses the secretion of melatonin, a sleep-promoting hormone, from the pineal gland [13–15]. This can interfere with sleep onset [13–15]. Thus, ST just before bedtime can be problematic.

This body of evidence suggests that not only the duration of ST but also ST before bedtime may affect obesity, physical activity, academic performance, and dry eyes in children. Therefore, in the current study, we examined the combined effects of ST duration and timing (just before bedtime) on these factors in elementary school children. To our knowledge, no study has examined multiple factors as objective variables for ST duration and timing in the same group of elementary school students. In this study, we aim to determine not only the influence of ST duration but also the novel combined effect from ST duration and timing.

## Methods

### Participants and the super diet education (Shokuiku) project

The Super Diet Education (Shokuiku) Project was a food education project supported by the Japanese Ministry of Education, Culture, Science and Technology. It was designed to develop programs to promote children’s dietary education in collaboration with various external organizations. The project’s overall aim was to contribute to a healthy lifestyle in school children and improve their health through nutrition education. In Japanese, “shoku” means diet, and “iku” means growth and education. In this project, a cross-sectional study was conducted on a cohort of elementary school children using a survey questionnaire. In total, 7419 children (6–12 years of age) from 18 elementary schools in Minato City, Japan participated in the survey during 2018–2019. Cases with missing data were excluded from the analysis. The study was approved by the Ethics Review Committee on Research with Human Subjects of Waseda University (application no. 2019–195).

### Questionnaire

Teachers at the schools explained the purpose of the study and distributed the questionnaires, and then children and their parents completed the questionnaires and returned them to the schools. Via the questionnaires, information was collected on gender, school year (1 to 6), learning ability, physical activity, ST duration and timing, and anthropometrics. Table 1 presents a summary of the question contents and response options. An additional file shows this in more detail [see Additional file 1].

**Table 1:** Question contents and response options for the explanatory variables and objective variables

	Explanatory variable	Content of question
Screen time	Duration	How much time do you spend per day playing on smartphones or computers, using communication applications, playing video games, or watching TV or videos?
		1. > 5 h; 2. 3 h to < 5 h; 3. 1 h to < 3 h; 4. < 1 h
	Timing	Just before you sleep, do you play on smartphones or computers, use communication applications, play video games, or watch TV or videos?
		1. Often; 2. Sometimes; 3. Rarely; 4. Never
	Objective variable	
Obesity	BMI percentiles	Calculated using height and weight
	Rohrer index	Calculated using height and weight
Physical activity	Weekly physical activity	In the last 7 days, how many days have you engaged in physical activities for more than 60 min?
		1. 0 days; 2. 1 day; 3. 2 days; 4. 3 days; 5. 4 days; 6. 5 days; 7. 6 days; 8. 7 days
Dry eyes	Dry eyes	Do you have dry eyes?
		1. Often; 2. Sometimes; 3. Rarely; 4. Never
Learning ability	Class	Do you understand the material presented in your classes at school?
		1. understand; 2. mostly understand; 3. slightly understand; 4. never understand
	Performance	Please describe your performance in classes at school (presentation, tests, etc.)
		1. perform very well; 2. perform in a satisfactory manner; 3. do not perform well; 4. cannot perform at all

### Obesity

The obesity variables consisted of the BMI percentiles and Rohrer index scores, which were assessed using self-reported height and weight. In 1997, the WHO and the International Obesity Task Force adopted BMI as a valid criterion for determining childhood obesity [16]. However, growth can affect BMI; therefore, it cannot be used in the same way in children as it can be in adults. As BMI tends to change considerably with age [17], the BMI percentiles were classified into two groups using age- and gender-appropriate charts [18] according to the following guidelines from Japan’s Ministry of Health, Labour and Welfare: “normal “(BMI ≤5th but <85th percentile) and “obese” (BMI ≥85th percentile). The Rohrer indexes were also divided into two groups: the “obese” group, with children who had Rohrer indices ≥145, and the “normal” group, with those who had Rohrer indices between 115 and 145. The Rohrer index score was calculated as shown below [19].

$$ \mathrm{Rohrer}\ \mathrm{index}=\mathrm{weight}\ \left(\mathrm{kg}\right)/\mathrm{height}\ {\left(\mathrm{cm}\right)}^3\times {10}^7 $$

### Physical activity

Large-scale population surveys, using a self-report questionnaire are the most feasible method for measuring physical activity [20, 21]. The WHO Health Behaviour in School-Aged Children (HBSC) survey is one of the most comprehensive sources of data on school-aged students’ physical activity levels [22]. The HBSC has been translated into Japanese (HBSC-J), and it has been shown to be valid [23]. In our questionnaire, we used the following item from the HBSC to assess how often participants engaged in moderate-to-vigorous physical exercise: “In the last 7 days, how many days have you engaged in physical activities for more than 60 minutes?” The responses to this question were categorized as follows: 1 for 0 days; 2 for 1 day; 3 for 2 days; 4 for 3 days; 5 for 4 days; 6 for 5 days; 7 for 6 days; and 8 for 7 days. The responses for weekly physical activities were divided into two groups: the “high physical activities” group with children who were above the median and the “low physical activities” group, with those who were below it.

### Dry eyes

The Dry Eye-Related Quality-of-Life Score (DEQS) questionnaire was created and validated in Japan [24]. We created the questionnaire items to assess dry eye symptoms based on the DEQS questionnaire. We asked the participants, “Do you have dry eyes?” The responses were on a scale from 1 to 4: 1, often; 2, sometimes; 3, rarely; and 4, never. “Dry eyes” responses were also divided into two groups: the “dry” group, with children who answered 1 or 2, and the “not dry” group, with those who answered 3 or 4.

### Learning ability

The study group consisted of “class,” and “performance,” which were divided into two groups. We asked the participants, “Do you understand the material presented in your classes at school?” The answers for “class” were categorized from 1 to 4: 1, understand; 2, mostly understand; 3, slightly understand; and 4, never understand. “Class” answers were then divided into two groups: the “understand” group, with children who answered 1 or 2, and the “do not understand” group, with those who answered 3 or 4. The questions used in this item were also used in a previous study [25]. In addition, we asked the participants, “Please describe your performance at school (in classes, on tests, etc.).” The answers for “performance” were categorized from 1 to 4: 1, perform very well; 2, perform in a satisfactory manner; 3, do not perform well; and 4, cannot perform at all. “Performance” answers were then divided into two groups: the “good” group, with children who answered 1 or 2, and the “poor” group, with those who answered 3 or 4. In Japan, where researchers’ access to children’s actual academic data is restricted, subjective learning ability is used as a feasible surrogate variable [26, 27]. Self-reported grades and actual grades have previously been reported to be generally accurate [28].

### ST duration and timing

Two items, the duration and timing of ST, were used as indicators of ST. We asked the participants, “How much time do you spend per day playing on smartphones or computers, using communication applications, playing video games, or watching TV or videos?” The responses for “duration of ST” were on a scale from 1 to 4: 1 to indicate > 5 h; 2 to indicate 3 h to < 5 h; 3 to indicate 1 h to < 3 h; and 4 to indicate < 1 h. The “duration of ST” responses were then divided into three groups: the “above 3 hours” group, with children who answered 1 or 2; the “1–3 h” group, with those who answered 3; and the “less than 1 hour” group, with those who answered 4. The current American Academy of Pediatrics guidelines recommend that children under 2 years of age should not spend any time using electronic media, while the ST of children over 2 years of age should be kept to less than 2 h per day [29, 30]. Therefore, 2 h is often used as a reference for ST. However, Minato City is implementing the “Minato-ku School Informatization Action Plan” and has been introducing electronic teaching materials in classes [31]. As a result, ST among Minato City elementary school students is increasing. Considering that headaches and sleep difficulties have been reported as after more than 3 h of ST [32], we used 3 h as the ST reference, which is 1 h more than the American Academy of Pediatrics guidelines. We asked the participants, “Just before you sleep, do you play on smartphones or computers, use communication applications, play video games, or watch TV or videos?” The responses for “timing of ST” were on a scale from 1 to 4: 1, often; 2, sometimes; 3, rarely; and 4, never. The “timing of ST” responses were then divided into two groups: the “yes” group, with children who answered 1 or 2, and the “no” group, with those who answered 3 or 4. Next, in order to examine differences in the influences of ST duration and timing, we used a combination of ST duration and timing as the explanatory variable (Table 2). For each objective variable, a logistic regression analysis comparing G1 and G2, G3 and G4, and G5 and G6 was performed.

**Table 2:** The combination of ST duration and timing

Group	ST duration			ST timing	
	Above 3 h	1–3 h	Less than 1 h	Yes	No
G1	〇			〇	
G2	〇				〇
G3		〇		〇	
G4		〇			〇
G5			〇	〇	
G6			〇		〇

### Statistical analyses

A chi-square test was performed to compare the sex and school year used as confounding factors by groups. The Wilcoxon test was used to compare height and weight by groups. The objective variables used in this study were as follows: “body mass index (BMI) percentiles” and the “Rohrer index” (for obesity), “weekly physical activities,” “dry eyes,” “class,” and “performance.” The explanatory variables were “duration of ST” and “timing of ST.” The ST in each group was examined using logistic regression analysis. First, we examined whether ST duration and timing were related to each objective variable. All variables were examined using a univariate model. Afterward, we performed multivariate logistic regression analyses for all variables that showed a significant difference in the univariate models. The odds ratios (ORs) and 95% confidence intervals (CIs) were calculated. The sample size was calculated to detect a medium effect [*f*^2^ (effect size) = 0.15]. A minimum sample size of 146 was required to have approximately 95% power to detect large effects at a significant level of 0.05 (G*Power, version 3.1.9.2, Universitat Kiel, Germany). All data were analyzed using predictive analytics software for Windows (Statistical Package for the Social Sciences; IBM Corp., Chicago, IL, USA); a *p* value of < 0.05 indicated statistical significance.

## Results

The characteristics of the “obese,” “physical activity,” “dry eyes,” and “learning ability” groups are presented in Tables 3, 4, 5 and 6. An analysis of the results of a questionnaire in which 6334 (85.38%) and 4683 (63.12%) of school children answered all items related to “BMI percentiles” and “Rohrer index”, respectively, was performed (Table 3). There were significant differences in gender and school year between the “normal” and “obese” groups. For the “weekly physical activities”, “dry eyes”, “class”, and “performance” items, 7048 (95.00%), 7041 (94.90%), 7026 (94.70%), and 7071 (95.31%) elementary school children, respectively, answered all questionnaire items and an analysis of the results was conducted (Tables 4, 5 and 6). There were significant differences in gender, school year, height, and weight between the “weekly physical activities” and “dry eyes” groups. Additionally, for the “performance”, between the “good” and “poor” groups, there were significant differences in gender, school year, and weight; however, there was no significant difference in height. For “class”, between the “understand” and “do not understand” groups, there were significant differences in gender, school year, and height; however, there was no significant difference in weight.

**Table 3:** Characteristics of the children in the obese group

		BMI percentiles						Rohrer index					
Item			Normal		Obese				Normal		Obese		
Age, mean (SE)			9.04	(0.023)	9.21	(0.063)			8.76	(0.023)	8.99	(0.069)	
		N	n	%	N	%	*P*-value^a^	N	N	%	N	%	*P*-value^a^
Gender	Boys	3346	2882	86.1	464	13.9	< 0.001	2582	2160	83.7	422	16.3	< 0.001
	Girls	2988	2744	91.8	244	8.2		2101	1886	89.8	215	10.2	
School year	1	1235	1126	91.2	109	8.8	0.021	1136	996	87.7	140	12.3	0.021
	2	1161	1039	89.5	122	10.5		945	833	88.1	112	11.9	
	3	1106	978	88.4	128	11.6		813	708	87.1	105	12.9	
	4	1080	949	87.9	131	12.1		730	626	85.8	104	14.2	
	5	989	857	86.7	132	13.3		617	511	82.8	106	17.2	
	6	763	677	88.7	86	11.3		442	372	84.2	70	15.8	

^a^Chi-square test

**Table 4:** Characteristics of the children in the physical activity group

		Weekly physical activities^c^					
Item			High		Low		
			Mean	SE	mean	SE	*P*-value^a^
Age			8.95	0.027	9.2	0.031	
Height			134.0	0.18	135.2	0.21	< 0.001
Weight			29.9	0.13	31.2	0.16	< 0.001
		N	N	%	n	%	*P*-value^b^
Gender	Boys	3619	2161	59.7	1458	40.3	< 0.001
	Girls	3429	1605	46.8	1824	53.2	
School year	1	1393	756	54.3	637	45.7	< 0.001
	2	1283	723	56.4	560	43.6	
	3	1218	705	57.9	513	42.1	
	4	1180	670	56.8	510	43.2	
	5	1111	560	50.4	551	49.6	
	6	863	352	40.8	511	59.2	

^a^Wilcoxon. ^b^Chi-square test

^c^The median number of physical activities per week was divided into two

**Table 5:** Characteristics of children with dry eyes

		Dry eyes					
Item			Not dry		Dry		
			mean	SE	mean	SE	*P*-value^a^
Age			8.98	0.023	9.44	0.048	
Height			134.1	0.16	136.8	0.32	< 0.001
Weight			30.1	0.11	31.9	0.24	< 0.001
		N	n	%	N	%	*P*-value^b^
Gender	Boys	3611	2921	80.9	690	19.1	0.25
	Girls	3430	2811	82.0	619	18.0	
School year	1	1385	1208	87.2	177	12.8	< 0.001
	2	1280	1073	83.8	207	16.2	
	3	1219	1007	82.6	212	17.4	
	4	1179	934	79.2	245	20.8	
	5	1114	870	78.1	244	21.9	
	6	864	640	74.1	224	25.9	

^a^Wilcoxon. ^b^Chi-square test

**Table 6:** Characteristics of the children in learning ability group

		Class						Performance					
Item			Understand		Do not understand				Good		Poor		
			mean	SE	mean	SE	*P*-value^a^		mean	SE	Mean	SE	*P*-value^a^
Age			9.08	0.021	8.71	0.103			9.03	0.022	9.26	0.053	
Height			134.7	0.14	132.3	0.72	< 0.001		134.5	0.15	135.1	0.37	0.090
Weight			30.5	0.10	30.5	0.59	0.089		30.2	0.11	31.7	0.29	< 0.001
		N	n	%	N	%	*P*-value^b^	N	n	%	N	%	*P*-value^b^
Gender	Boys	3594	3414	95.0	180	5.0	0.009	3623	2962	81.8	601	16.6	0.001
	Girls	3432	3304	96.3	128	3.7		3438	3022	87.9	476	13.8	
School year	1	1389	1305	94.0	84	6.0	0.017	1394	1226	87.9	168	12.1	< 0.001
	2	1273	1213	95.3	60	4.7		1288	1109	86.1	179	13.9	
	3	1218	1171	96.1	48	3.9		1223	1052	86.0	171	14.0	
	4	1174	1132	96.4	42	3.6		1182	976	82.6	206	17.4	
	5	1114	1070	96.1	44	3.9		1113	909	81.7	204	18.3	
	6	858	827	96.4	31	3.6		861	712	82.7	149	17.3	

^a^Wilcoxon

^b^Chi-square test

The results of the multivariate analysis for each objective variable are presented in Table 7. The duration (1 h to < 3 h: OR = 0.61, 95% CI = 0.50–0.74; < 1 h: OR = 0.42, 95% CI = 0.33–0.54) and timing of ST (OR = 0.78, 95% CI 0.65–0.93) were positively correlated with the “BMI percentiles.” These results show that the participants with shorter ST durations per day were more likely to be in the “normal” group. In addition, the results show that those who did not have ST before bedtime were more likely to be in the “normal” group than those who did. The duration (1 h to < 3 h: OR = 0.65, 95% CI = 0.52–0.81; < 1 h: OR = 0.43, 95% CI = 0.32–0.57) and timing of ST (OR = 0.73, 95% CI = 0.60–0.90) were positively correlated with the “Rohrer index” item. These results show that the participants with shorter ST durations per day were more likely to be in the “normal” group. In addition, the results show that those who did not have ST before bedtime were more likely to be in the “normal” group.

**Table 7:** Results of logistic regression analysis of ST duration and timing

		Obesity		Physical activity	Dry eyes	Learning ability	
		BMI percentiles	Rohrer index	Weekly physical activity^b^	Dry eyes	Class	Performance
		OR^a^	OR^a^	OR^a^	OR^a^	OR^a^	OR^a^
		95%Cl	95%Cl	95%Cl	95%Cl	95%Cl	95%Cl
Duration of ST	Above 3 h	1	1	1	1	1	1
	1-3 h	0.61^***^	0.65^***^	1.19^*^	1.15	2.24^***^	1.67^***^
		0.50–0.74	0.52–0.81	1.03–1.37	0.97–1.36	1.71–2.94	1.41–1.98
	Less than 1 h	0.42^***^	0.43^***^	1.27^**^	1.19	3.93^***^	2.4^***^
		0.33–0.54	0.32–0.57	1.08–1.48	0.98–1.45	2.70–5.71	1.95–2.96
Timing of ST^c^	No/(Yes)	0.78^**^	0.73^**^	1.06	1.31^***^	1.43^**^	1.55^***^
		0.65–0.93	0.60–0.90	0.95–1.17	1.15–1.50	1.10–1.86	1.33–1.79

^***^p < 0.001, ^**^p < 0.01, ^*^p < 0.05

^a^*OR* odds ratio, *95% CI* 95% confidence interval

^b^The median number of physical activities per week was divided into two

^c^Whether there was ST just before bedtime

The duration of ST (1 h to < 3 h: OR = 1.19, 95% CI = 1.03–1.37; < 1 h: OR = 1.27, 95% CI = 1.08–1.48) was negatively correlated with the “weekly physical activities” item, while ST timing was not. These results show that the participants with shorter durations of ST per day participated in more physical activities.

The ST timing (OR = 1.31, 95% CI = 1.15–1.50) was negatively correlated with the “dry eyes” item, while ST duration was not. These results show that students who did not have ST just before bedtime were less likely to have dry eyes compared to those who did.

The duration (1 h to < 3 h: OR = 2.24, 95% CI = 1.71–2.94; < 1 h: OR = 3.93, 95% CI = 2.70–5.71) and timing of ST (OR = 1.43, 95% CI = 1.10–1.86) were associated with the “class” item. These results show that the participants with shorter ST durations per day had a better understanding of the material presented in their classes. In addition, results show that those who did not have ST before bedtime were more likely to understand the material presented in their classes than those who did. For the “performance” item, the duration (1 h to < 3 h: OR = 1.67, 95% CI = 1.41–1.98; < 1 h: OR = 2.40, 95% CI = 1.95–2.96) and timing of ST (OR = 1.55, 95% CI = 1.33–1.79) were negatively correlated with the academic performance of the participants. These results show that the participants with shorter durations of ST per day showed good performance. In addition, it showed that children who had no ST just before bedtime were more likely to have good performance than those who did.

### Combination of the duration and timing of ST

In this study, we had six combination groups (G1, G2, G3, G4, G5, and G6) (Table 2)**.** We performed logistic regression analysis to compare G1 and G2, G3 and G4, and G5 and G6 regarding each item. The results are presented in Table 8. For the “BMI percentiles” item, the differences between G4 and G3 (OR = 0.72; 95% CI = 0.57–0.90) was associated with being in the “normal” group. In the “Rohrer index” item, the differences between G4 and G3 (OR = 0.76; 95% CI = 0.60–0.97) was associated with being in the “normal” group. In the “weekly physical activities” item, no predominant association in any combination group was observed. Furthermore, the differences between G4 and G3 (OR = 1.36; 95% CI = 1.14–1.63) and G6 and G5 (OR = 1.38; 95% CI = 1.08–1.77) were associated with “not dry eyes.” Regarding the “class” item, no predominant association in any combination group was noted. The differences between G2 and G1 (OR = 1.85; 95% CI = 1.26–2.72), G4 and G3 (OR = 1.53; 95% CI = 1.26–1.85) and G6 and G5 (OR = 1.42; 95% CI = 1.05–1.91) were associated with good grades. These results show that the timing of ST had a greater impact on dry eye symptoms, while the duration of ST had a greater effect on academic performance.

**Table 8:** Results of the combination of ST duration and timing

	BMI percentiles	Rohrer index	Weekly physical activities^b^	Dry eyes	Class	Performance
	OR^a^	OR^a^	OR^a^	OR^a^	OR^a^	OR^a^
	95%Cl	95%Cl	95%Cl	95%Cl	95%Cl	95%Cl
G1^c^ × G2^c^	0.73	0.64	1.09	1.04	1.59	1.85^**^
	0.47–1.12	0.40–1.05	0.81–1.48	0.72–1.49	0.88–2.87	1.26–2.72
G3^C^ × G4^C^	0.72^**^	0.76^*^	1.09	1.36^***^	1.37	1.53^***^
	0.57–0.90	0.60–0.97	0.95–1.25	1.14–1.63	0.97–1.93	1.26–1.85
G5^C^ × G6^C^	1.05	0.73	0.99	1.38^*^	1.41	1.42^*^
	0.71–1.56	0.49–1.09	0.81–1.20	1.08–1.77	0.78–2.56	1.05–1.91

^***^p < 0.001, ^**^p < 0.01, ^*^p < 0.05

^a^*OR* odds ratio, *95% CI* 95% confidence interval

^b^The median number of physical activities per week was divided into two

^c^Divided into G1 to G6 based on the combination of ST duration and timing (Please refer to Table 2)

## Discussion

### Main results

This study surveyed elementary school children from Minato City to examine the effects of ST duration and timing on obesity, physical activity, dry eyes, and learning ability. The relationships between ST duration and BMI percentile, Rohrer index, weekly physical activity, class, and performance were statistically significant. Furthermore, the relationships between ST timing and BMI percentile, Rohrer index, dry eyes, class, and performance were also statistically significant. In addition, ST timing greatly affected dry eye symptoms, whereas ST duration greatly affected academic performance.

### Relationship between ST duration and obesity, physical activity, academic performance, dry eyes

In the present study, children with shorter ST durations were more likely to have normal body weight, higher physical activity, better understanding of the material presented in their classes, and better academic performance.

The relationship between screen media exposure and obesity has been extensively studied. For example, many studies have reported a relationship between watching TV and development of obesity [33–35]. A possible cause for the obesity associated with TV viewing is increased caloric intake while watching TV. Watching TV while eating a meal may increase caloric intake by delaying satiety during meals or by reducing satiety signals from previously consumed food; it may also divert attention from the habitual control of food intake [36]. In addition, longer ST durations are associated with increased sedentary behavior and decreased physical activity, which may be related to obesity. It has been previously reported that children with longer STs have greater obesity and adiposity [37]. Moreover, longer durations of ST, especially due to watching TV, are associated with decreased physical fitness [3, 38] and decreased muscle strength, regardless of the physical activity level [39]. Those with longer TV viewing time spend less time in club sports, which may indicate less involvement in overall physical activity [38]. The findings from these studies are consistent with those of our present study that individuals with shorter ST durations spent more time participating in physical activities.

In addition, previous studies have shown that longer ST durations detract from time spent on academic activities such as studying and doing homework [40], and this can cause learning and attention deficits and negative attitudes toward attending school [41]. Another study reported that Japanese children with shorter ST durations were more likely to have high academic performance, regardless of their physical activity level [42]. In addition, spending on ST for more than 2 h per day was negatively associated with academic achievement in school-age children [3]. Taken together, these studies suggest that longer ST durations can influence individual behavioral styles that impinge on academic understanding and performance and contribute to poor grades.

Because the influence of ST through smartphones and tablets is included in this survey, ST other than watching TV may be significantly associated with obesity, physical activity, dry eyes, and academic performance.

### Relationship between ST timing and obesity, physical activity, academic performance, dry eyes

Our results showed that compared to those who had ST, those who did not have ST just before bedtime were more likely to have normal body weight, had no dry eyes, understood the material presented in their classes, and had better academic performance. This may be because late-night ST contributes to evening chronotype behavior [12], and late-night snack consumption is associated with a higher risk of developing obesity and metabolic diseases [43, 44].

As mentioned above, it is believed that ST immediately before bedtime leads to sleep deprivation in children, and studies have reported that sleep deprivation is associated with poor grades [45]. Our results confirmed this hypothesis. Sleep deprivation affects memory retention, increases erroneous memory formation [46], and is associated with lack of judgment and attention [47]. Therefore, sleep deprivation from ST immediately before bedtime could affect attitudes toward learning, learning comprehension, and, ultimately, overall academic achievement.

Moreover, digital device use has been correlated with dry eye symptoms [48] and tear film instability [49]. The interblink interval and tear film instability increase during highly focused work [9–11]. Thus, in the current study, ST immediately before bedtime may be associated with focused ST exposure. Other studies reported that blue light emitted from smart mobile device screens causes eye fatigue [50, 51] and that dry eyes are associated with sleep quality [52]. Hence, ST before bedtime can detract from sleep quality and lead to dry eyes.

### Influence of the combination of ST duration and ST timing

Jointly investigating ST duration and timing showed that ST timing had a greater effect than ST duration on dry eye symptoms. Thus, the timing of ST has a significant impact on dry eyes. Currently, we do not know the mechanism behind this, but several possibilities exist. Late-night ST may lead to dry eye symptoms as tear secretion follows the circadian rhythm, with low levels at 21:00 [53]. Further, late-night ST may promote sympathetic activity, and activation of the sympathetic nervous system is known to decrease tear secretion [54]. Another possibility is related to focused nighttime ST exposure, considering that children may be using digital devices without parental supervision at night. Focused ST exposure causes incomplete blinks and tear film instability [9–11], and these factors contribute to the risk of dry eyes [55–57]. Regarding academic performance, our study has shown that the ST duration has a greater effect than ST timing, and longer ST durations detract more from academic performance. However, the mechanism of the ST duration and timing effect is not yet clear. Spending more than 2 h per day in front of the screen was negatively associated with academic achievement among school-age children [3]. Thus, having sufficient study time in the afternoon, early evening, and late evening may be a very important factor in academic achievement. Our results call attention to the different effects of ST dependency (duration and/or timing) on individual areas. Blue light exposure before bedtime causes disturbances of the circadian clock, sleep length, energy metabolism, and academic performance [15]. Among the visible light wavelengths, short wavelengths perceived as blue can strongly affect the phase delay of the circadian rhythm and suppress melatonin [13–15]. Most digital devices emit blue light. Thus, it would be preferable to avoid using digital devices before bedtime, as it could significantly influence sleep quality and the circadian rhythm [15]. Many adolescents exhibit an evening chronotype from exposure to nighttime blue light, and their biological and social rhythms become misaligned. This chronotype can result in sleep disturbances as well as fatigue, daytime sleepiness, behavioral problems, and poor academic achievement, among other negative outcomes [15]. Therefore, parents, school teachers, and leading social media firms should strive to reduce children’s late-night ST.

### Study strengths and limitations

In this study, we investigated ST effects on multiple objective variables using the same group of elementary school children. To our knowledge, no studies have examined the effect of the duration and timing of ST on obesity, physical activity, dry eyes, and learning ability in the same group of elementary school children. In addition, by combining the duration and timing of ST, we were able to show not only the effect of ST duration, which has already been discussed in the literature, but also that of ST timing (just before bedtime). The combined analysis of ST duration and timing is a strength of this study.

However, our study has several limitations. First, the surveys failed to reflect the actual lifestyles and anthropometric data of the participants. Children may have exaggerated or downplayed aspects of their lifestyles based on social expectations. Second, the study relies on children’s answers, which may introduce errors due to their varying interpretations of the questions. Third, because the study focused only on elementary school students, the results may not be applicable to middle school, high school, and college students along with working adults and hence, may not be generalizable. Therefore, it may be necessary to expand the range of participants in future studies. Fourth, the area covered by this study was Minato City, Tokyo, Japan, and it is unclear whether the same results would be obtained in other areas. Therefore, it may be necessary to expand the target area in future studies. Fifth, the results on “dry eyes” obtained in this study are for assessing symptoms, not for diagnosing dry eye. Therefore, it will be necessary to examine them in greater detail using dry eye disease diagnosis indicators. Finally, the validity of the questionnaire used on children in this study has not yet been verified. However, it was created with reference to a questionnaire for adults that has been verified in previous studies; therefore, it is considered that the validity of the result is likely to be guaranteed.

## Conclusion

In conclusion, our study demonstrated that ST immediately before bedtime increases the risk of dry eyes, and longer ST duration can contribute to reduction in academic performance. In addition, our findings indicate that not only the duration but also the timing of ST have important ramifications.

## Supplementary Information

**Additional file 1.** Questionnaire on the Shokuiku.

## Data Availability

The datasets for the current study are available from the corresponding author on reasonable request.

## Abbreviations

ST: Screen time
BMI: Body mass index
WHO: World health organization
HBSC: Behaviour in School-Aged Children
DEQS: Dry Eye-Related Quality-of-Life Score
ORs: Odds ratios
Cis: Confidence intervals
SE: Standard Error
