# Supplementary material for: Screen time duration and timing: effects on obesity, physical activity, dry eyes, and learning ability in elementary school children
Source: BMC Public Health. 2021 Feb 28;21:422. doi: 10.1186/s12889-021-10484-7 (PMC7916284; doi:10.1186/s12889-021-10484-7)
Supplement: Supplementary file 1 — Additional file 1. Questionnaire on the Shokuiku. [file 12889_2021_10484_MOESM1_ESM.pdf]

Questionnaire on the Shokuiku

Q1. How old are you?

( ) years

Q2. Which is your gender? (Circle only one)

1.Male 2. Female

Q3. Which grade are you? (Circle only one)

1 2 3 4 5 6

Q4. How tall are you?

( ) cm

Q5. How much do you weigh?

( ) kg

Q6. How much time do you spend per day playing on smartphones or computers, using communication applications, playing video games, or watching TV or videos? (Circle only one)

1. > 5 h 2. 3h to < 5h 3. 1h to < 3h 4. < 1h

Q7. Just before you sleep, do you play on smartphones or computers, use communication applications, play video games, or watch TV or videos? (Circle only one)

1. Often 2. Sometimes 3. Rarely 4. Never

Q8. In the last 7 days, how many days have you engaged in physical activities for more than 60 minutes? (Circle only one)

1. 0 days 2. 1 day 3. 2 days 4. 3 days
5. 4 days 6. 5 days 7. 6 days 8. 7 days

Q9. Do you have dry eyes? (Circle only one)

1. Often 2. Sometimes 3. Rarely 4. Never

Q10. Do you understand the material presented in your classes at school? (Circle only one)

1. understand 2. mostly understand 3. slightly understand 4. never understand

Q11. Please describe your performance in classes at school (presentation, tests, etc.) (Circle only one)

1. perform very well 2. perform in a satisfactory manner
3. do not perform well 4. cannot perform at all
